# Apple Supplementation Improves Hemodynamic Parameter and Attenuates Atherosclerosis in High-Fat Diet-Fed Apolipoprotein E-Knockout Mice

**DOI:** 10.3390/biomedicines8110495

**Published:** 2020-11-12

**Authors:** Raffaella Soleti, Charlotte Trenteseaux, Lionel Fizanne, Marine Coué, Gregory Hilairet, Fatima Kasbi-Chadli, Patricia Mallegol, Julien Chaigneau, Jerome Boursier, Michel Krempf, Mathilde Orsel, Khadija Ouguerram, Ramaroson Andriantsitohaina

**Affiliations:** 1 SOPAM, U1063, INSERM, UNIV Angers, SFR ICAT, 49 800 Angers, France; gregory.hilairet@univ-angers.fr (G.H.); patricia.mallegol@univ-angers.fr (P.M.); 2 UMR 1280 Physiopathologie des Adaptations Nutritionnelles (PhAN), INRAE, Université de Nantes, 44 093 Nantes, France; charlotte.trenteseaux@gmail.com (C.T.); marine.coue@univ-nantes.fr (M.C.); Fatima.Chadli-Kasbi@univ-nantes.fr (F.K.-C.); Michel.Krempf@univ-nantes.fr (M.K.); khadija.ouguerram@univ-nantes.fr (K.O.); 3 Centre de Recherche en Nutrition Humaine Ouest, 44 000 Nantes, France; 4 EA 3859, Hémodynamique, Interaction Fibrose et Invasivité Tumorales Hépatiques (HIFIH), 49 933 Angers, France; lionel.fizanne@univ-angers.fr (L.F.); julien.chaigneau@univ-angers.fr (J.C.); JeBoursier@chu-angers.fr (J.B.); 5 IRHS-UMR1345, Université d’Angers, INRAE, Institut Agro, SFR 4207 QuaSaV, 49071 Beaucouzé, France; mathilde.orsel-baldwin@inrae.fr

**Keywords:** apple supplementation, hemodynamic parameter, atherosclerosis, high fat diet, ApoE deficient mice

## Abstract

Epidemiological studies describe the association between apple consumption and improved cardiovascular and metabolic dysfunction. Our recent multiparametric screening on cellular model studies has shown that apples exhibit vascular tropism including Granny Smith (GS) variety independently of the storage condition. The present study aimed to evaluate the cardiovascular and metabolic protection of supplementation of GS variety after storage in classic cold (GSCC) and extreme ultra-low oxygen conditions (GSXO) in the apolipoprotein E-deficient 8-week-old mice fed with high fat diet for 14 weeks. Supplementation with GSCC and GXO decreases circulating triglycerides, the expression of genes involved in lipogenesis, without change in cholesterol and glucose concentrations and HOMA-IR. Only GSXO supplementation ameliorates body weight gain, insulin level, and HDL/LDL ratio. GSXO supplementation does not modify cardiac parameters; while supplementation with GSCC decreases heart rate and improves cardiac output. Interestingly, GSCC and GSXO reduce systolic and diastolic blood pressure with a differential time course of action. These effects are associated with substantial decrease of atherosclerotic lesions. These data reinforce the knowledge about the vascular tropism of apple supplementation and underscore their ability to improve both cardiovascular and metabolic alterations in a mouse model of atherosclerosis.

## 1. Introduction

Obesity is a rapidly growing problem that is reaching epidemic proportions worldwide, and is associated with an increased risk of cardiovascular and metabolic diseases. Obesity-related diseases result from dysregulated lifestyle, including diet and physical activity. Pharmacological basis of epidemiological studies supports the hypothesis that intake of diet rich in fruits and vegetables induces a greater reduction in cardiovascular risk and metabolic disorders. Among fruits, apple is a source rich in polyphenols, fibers, proteins, carbohydrates, vitamins, and minerals [1]. Several studies evidence that apple intake not only improves vascular function [2], lipid metabolism [3], weight management [4], and inflammation [5,6], but it has also been associated to a lower risk for diabetes [7].

In contrast to a significant number of preclinical studies focusing on a particular apple compound and/or juice derivative, few studies evaluate the consumption of whole apple fruit. Indeed, intake of whole apple decreases lipids including cholesterol, triglycerides (TG), and low-density lipoprotein cholesterol in humans [8,9]. In a multiparametric screening of different varieties of apple fruits under different storage conditions, we suggested the potential use of some whole apple extracts as effective food supplements or nutraceuticals for the prevention and/or management of cardiovascular and metabolic diseases [10]. Indeed, apple flesh extract from Granny Smith (GS) variety after 4 months cold storage in classic cold conditions (GSCC) and extreme ultra-low oxygen conditions (GSXO) emerge as a good candidate. In cell models associated with vascular and metabolic dysfunctions GS under both storage conditions displayed vascular and hepatic tropism [10]. The present study aimed to evaluate the effect of either CC or XO GS supplementation under both described storage conditions in a model of cardiovascular and lifestyle-related cardiometabolic diseases, the apolipoprotein E-deficient (ApoE-/-) mice fed with high fat diet (HFD).

The present investigation focuses on a set of cardiometabolic features that are known to increase cardiovascular risks: body weight, glucose and lipid regulation, hepatic, cardiac, and hemodynamic parameters as well as atherosclerotic lesions.

## 2. Experimental Section

### 2.1. Products

‘Granny Smith’ apple fruit (Malus domestica Borkh.) were harvested from commercially run orchards at the Station Experimental de La Morinière (30 ha estate, Saint Epain, France) and were stored as previously described [10]. GS flesh samples under cold condition (CC) (0.5 °C) and extra low oxygen (XO) (0.5 °C, 0.8% O_2_, 0.8% CO_2_) were selected upon in vitro screening [10].

Flesh samples were included by SAFE (Augy, France) into a diet supplied in lyophilized form at the concentration of 3.33 g/kg standard diet (SD) and HFD deficient in choline. Diet composition is detailed on Table 1.

### 2.2. Determination of Polyphenol Content in GS

Simple polyphenols, including monomeric catechins, low molecular weight procyanidins, hydroxycinnamic acids, flavonols, dihydrochalcones, and anthocyanins, were extracted and quantified as previously described [10].

### 2.3. Ethics Statement

All procedures were carried out simultaneously in the laboratories of Universities of Angers and Nantes in accordance with the guidelines and authorization with French Ministry of Agriculture regulations based on European Community and were approved by the local ethics committee “Comité d’éthique en expérimentation animale Pays de la Loire”; Apafis#320-2015031314466612_v2 and Apafis#1687-2015060312546655.

### 2.4. Animals

Eight-week old male and female ApoE-/- mice were obtained from Charles River (L’Arbresle, France) and from the animal housing unit of the University of Angers (Angers, France) and the University of Nantes (Nantes, France). The animals were housed in a controlled environment room, with light/dark cycle conditions (12-h light/12-h dark) and ambient temperature at 23 °C ± 2 °C.

The animals received diet and water ad libitum. The mice were randomly divided into four groups. The control groups (n from 6 to 10) were fed with a SD or HFD. Two other groups (n from 6 to 10) were fed with a HFD diet containing apple samples (HFD GSCC and HFD GSXO) for 14 weeks. The duration of HFD (14 weeks) was based on the period of time necessary to induce the metabolic alterations in the ApoE-/- mice [11,12].

Mouse weight was measured weekly. Blood pressure and heart rate were measured four times (at the beginning, after one and two months and at the end of the protocol). One day before euthanasia, cardiac parameters were evaluated by echocardiography. At the end of protocol, mice were maintained in fasting conditions overnight (≈ 12 h before euthanasia) and underwent glucose tolerance test. Then they were euthanized, blood was collected, the visceral and subcutaneous fat, liver, heart, and aorta were removed for histological and biochemical analysis. The adiposity was calculated as the total adipose tissue weight (the sum of the visceral and subcutaneous adipose tissues) vs. total body weight. Fragments of tissue of each animal were frozen at −80 °C for further analysis.

### 2.5. Blood Pressure and Heart Rate Measurements

Noninvasive blood pressure (systolic, diastolic, and mean) and heart rate were measured using tail-cuff method with BP-2000 blood pressure analysis system (Bioseb, Vitrolles, France). Mice were trained everyday over a period of a week to get accustomed to the device prior the beginning of experimental protocol. Ten successive measurements were recorded and averaged.

### 2.6. Echocardiography 

Transthoracic echocardiography was performed on anesthetized (1.5% isoflurane) mice using the Vevo 770 ultrasound echography from FUJIFILM VisualSonics (Toronto, ON, Canada) with a 30 MHz imaging transducer. Parasternal short-axis images were obtained in M-mode. Functional and structural modifications were evaluated as previously described [13].

### 2.7. Biochemical Parameters

Plasma samples were obtained by blood centrifugation at 900 × *g* during 10 min at 4 °C, frozen in liquid nitrogen, and stored at −80 °C until the dosage. Fasting glucose, triglycerides (TG), total cholesterol, low density lipoprotein (LDL)-cholesterol and high-density lipoprotein (HDL)-cholesterol were measured using Konelab™ 20 Clinical Chemistry Analyzer (Thermo Scientific™, Waltham, MA, USA). Plasma insulin was determined by enzyme-linked immunosorbent assay (Merck-Millipore, Germany). The homeostasis model for insulin resistance (HOMA-IR) was calculated via the following formula: fasting blood glucose × fasting plasma insulin/22.5.

### 2.8. Hepatic Secretion of very Low Density Lipoprotein (VLDL) TG

To measure hepatic TG production rate, mice were intraperitoneally injected with 500 mg/kg of Triton-W1339 (Sigma) that inhibited lipoprotein lipase (LPL) [14]. Blood samples were collected from the tail vein before and at 120 and 300 min after injection. TG concentration was determined with the enzymatic assay (Dyasis, Grabels France). TG production rate for individual mice was therefore calculated using linear increment between the baseline value and 300 min [15] and expressed as mg/dL/h.

### 2.9. RNA Extraction and Real-Time Quantitative Polymerase Chain Reaction (RT-qPCR)

Total tissue RNA extraction was processed using TRizol reagent (Life Technologies, Saint Aubin, France) according to manufacturer instructions. After reverse transcription of 1 µg total RNA realized with SuperScript III Reverse Transcriptase (Life Technologies, Saint Aubin, France) and DNAse treatment (Promega, Charbonnières-les-Bains, France), samples were analyzed on a Bio-Rad CFX Manager system (Bio-Rad, Marnes-la-Coquette, France). All primer sequences (Eurofins, Nantes, France) were available on request. All reactions were performed at least in triplicate, and Cyclophilin RNA amplification was used as a reference. In all PCR assays and for each primer set, expression of a control cDNA was included as inter-run calibrator. Expression data were normalized by the 2 (DCt) method using Tata-box binding protein (Tbp) as internal control.

### 2.10. Atherosclerotic Lesions Analysis

To study atherosclerosis progression, mice were euthanized under anesthesia with isoflurane at 22 weeks old (n = 6–7 per group). Physiologic sodium chloride solution was injected into the systemic circulation. The entire aorta between the heart and iliac arteries was dissected. The peripheral fat of the upper aorta was removed under a stereo microscope. The heart, the aortic arch and the entire arterial tree (thoracic and abdominal) were separated.

### 2.11. Atherosclerotic Lesions in Aortic Root

The heart and 2–3mm long aortic arch were frozen by isopentane −80 °C embedding medium for serial 10 μm-thick cryosectioning. Serial cross-sections of three valve leaflets were sectioned (from 200 to 250 μm, from 400 to 450 μm, from 600 to 650 μm, and from 800 to 850 μm between the base of the heart and aortic arch). All the sequential sections were stored at −80 °C until use. Then, the four glass slides were stained with Oil red O and counterstaining with hematoxylin. Images of all stained slides were captured using a digital slide scanner (Nanozoomer Hamamatsu 2.0 HT, Japan). Quantitative analysis of neutral lipid-stained lesions was performed using image analysis software ‘Nanozoomer Digital Pathology View software’. (http://rsb.info.nih.gov/ij/). Lesions in cross sections through the aortic root were traced during morphometric analysis. Results were expressed in μm^2^ as the average of neutral lipid area in the lesion per section, as previously described [16].

### 2.12. Liver Histology

Liver lobe 1 and 2 slices were fixed in 4% paraformaldehyde for 24 h before paraffin-embedding. Then 5 µm thick sections were stained with hematoxylin-eosin-saffron or 0.1% picrosirius red solution. Finally, the entire stained specimen was analyzed to quantify area of steatosis and fibrosis as previously described [17].

### 2.13. Statistical Analysis

Data were represented as mean ± standard error of the mean (SEM), n represents the number of animals (6–10). Statistical analyses were performed by the analysis of variance (ANOVA), or Mann–Whitney U tests and subsequent Bonferroni/Sidak post hoc test. *p* < 0.05 is considered to be statistically significant. All analyses were performed with GraphPad Prism 6 software (GraphPad Software, San Diego, CA, USA).

## 3. Results

### 3.1. Effects of Apple Supplementation on Body Weight Gain

**Figure 1:**
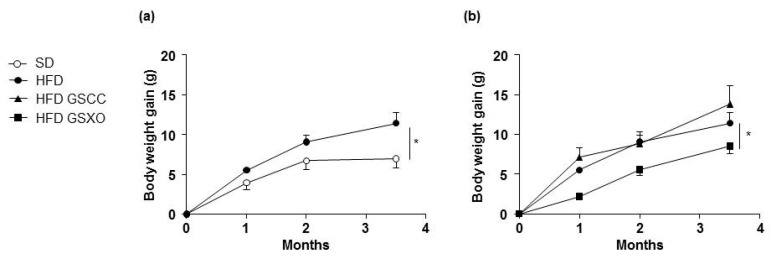
Effect of diet and apple supplementation on body weight of ApoE-/- mice. The evolution of body weight gain (**a**,**b**) of mice receiving standard diet (SD), high fat diet (HDF) (**a**), or HFD containing GSCC and GSXO (**b**) for 14 weeks. The data were expressed as the mean ± SEM. * *p* < 0.05.

HFD induced a time-dependent increase in body weight gain compared to SD-fed mice (Figure 1a). Interestingly, GSXO but not GSCC supplementation prevented weight gain in HFD-fed mice (Figure 1b).

### 3.2. Effects of Apple Supplementation on Glucose and Lipid Parameters

Blood glucose levels were not significantly different among the four groups (Table 1). Plasma insulin level was not significantly different between SD and HFD mice. GSXO but not GSCC supplementation increased insulin concentration when compared to HFD. HOMA-IR index, an indicator of insulin resistance, was not significantly different in all groups of mice (Table 2).

**Figure 2:**
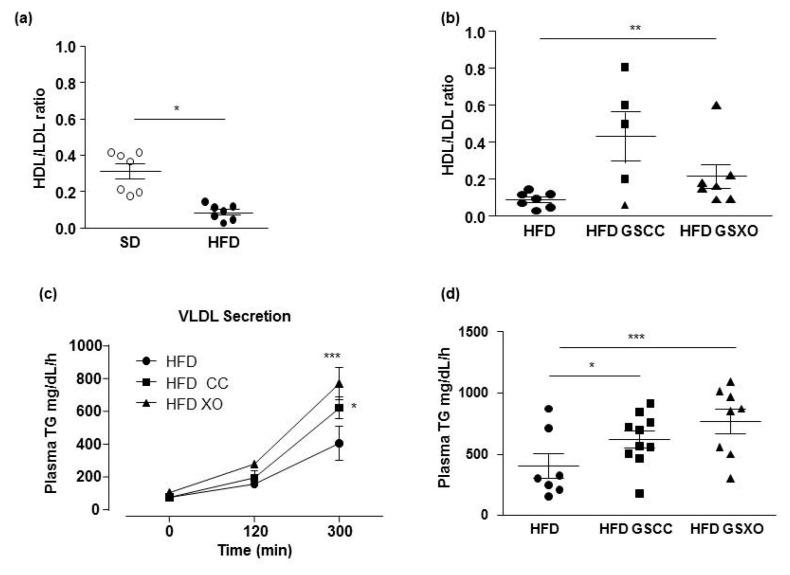
Effect of diet and apple supplementation on lipid parameters in ApoE-/- mice. Ratio of HDL- to LDL-cholesterol (**a**,**b**), very low density lipoprotein (VLDL) secretion (**c**) and VLDL secretion after 3 h (**d**) in mice receiving standard diet (SD; white circle), high fat diet (HFD; black circle), and HFD supplemented with apple for 14 weeks (black square and black triangle for HFD supplemented with GSCC and GSXO, respectively). The data were expressed as the mean ± SEM. Statistical analyses were performed by Mann–Whitney U tests, (**a**) one-way ANOVA, and post hoc analyses followed by Sidak correction (**b**–**d**), * *p* < 0.05, ** *p* < 0.01, *** *p* < 0.001.

HFD increased the plasma levels of TG compared to SD-fed mice (Table 1). Both GSCC and GSXO supplementations decreased TG levels significantly. No differences were observed in cholesterol concentration between the four groups of mice (Table 1). The HDL/LDL ratio was decreased by HFD (Figure 2a). Supplementation with GSXO but not GSCC significantly improved the HDL/LDL ratio (Figure 2b).

In HFD groups, hepatic secretion rate was decreased (53.0 ± 40.6 mg/g/h) compared to SD (109.8 ± 58.1 mg/dL/h; *p* < 0.05, data not shown). Five hours after injection of Triton-1339, the hepatic TG secretion rate was enhanced with GSCC and GSXO supplementations (621.6 ± 67.7; *p* < 0.001 and 770.4 ± 99.6 mg/dL/h; *p* < 0.05, respectively) compared to HFD group (404.5 ± 104 mg/dL/h) (Figure 2c,d).

### 3.3. Effects of Apple Supplementation on Liver

**Figure 3:**
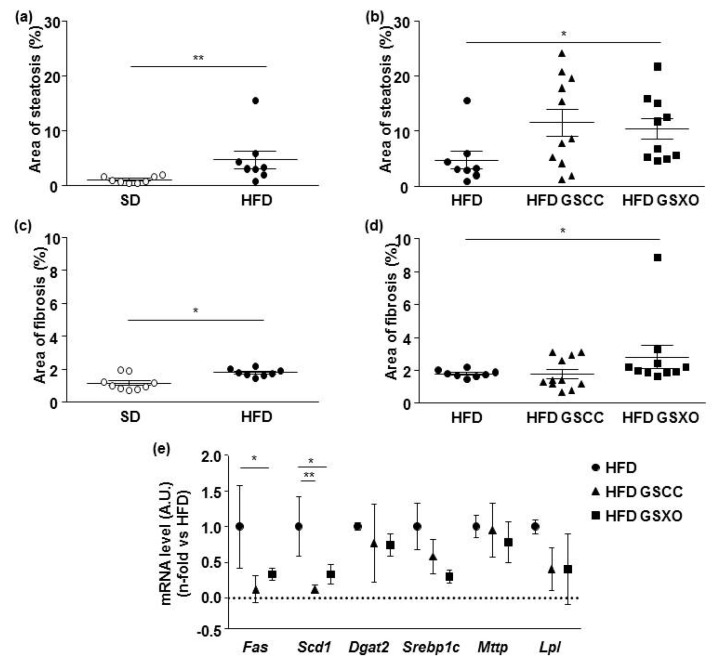
Effect of diet and apple supplementation on liver of ApoE-/- mice. Steatosis (**a**,**b**) and fibrosis (**c**,**d**) area in mice receiving standard diet (SD, with circle), high fat diet (HFD, black circle), and HFD supplemented with apple for 14 weeks (black square and black triangle for HFD supplemented with GSCC and GSXO, respectively). (**e**) Hepatic relative expression of mRNA levels of several genes involved in lipogenesis: fatty acid synthase (*Fas*), stearoyl-coenzyme A desaturase-1 (*Scd1*); diacylglycerol O-acyltransferase 2 (*Dgat2*); sterol regulatory element-binding transcription factor 1 (*Srebp1c*); microsomal TG transfer protein (*Mttp*); lipoprotein lipase (*Lpl*). The data were expressed as the mean ± SEM. * *p* < 0.05, ** *p* < 0.01.

Hepatic steatosis and fibrosis area were increased by HFD (Figure 3a,c). Supplementation with GSCC did not modify these parameters although GSXO weakly increased them (Figure 3b,d).

The relative expression of mRNA levels for some lipogenic enzyme genes (i.e., diacylglycerol O-acyltransferase 2, *Dgat;* sterol regulatory element-binding transcription factor 1, *Srebp1c*; microsomal TG transfer protein; *Mttp*; *Lpl*) were not affected by apple supplementation (Figure 3e). By contrast, the relative expression of the fatty acid synthase (*Fas*) gene was decreased in GSXO groups compared HFD mice (Figure 3e). Additionally, both apples decreased the relative expression of stearoyl-coenzyme A desaturase-1 (*Scd1*) (Figure 3a).

### 3.4. Effects of Apple on Heart Function

Structural cardiac parameters measured by echocardiography (Table 3) were not affected by the different treatments throughout the study. Only GSCC supplementation significantly improved the cardiac output.

### 3.5. Effects of Apple Supplementation on Blood Pressure and Heart Rate

**Figure 4:**
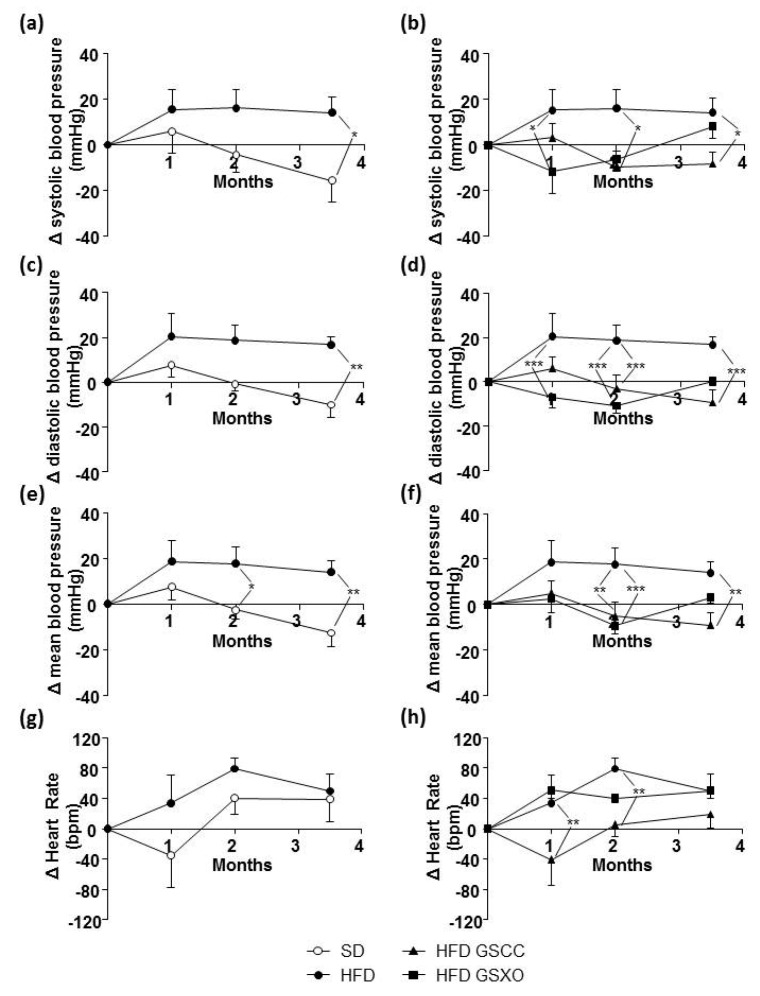
Effect of diet and apple supplementation on hemodynamic parameter of ApoE-/- mice. Systolic (**a**,**b**), diastolic (**c**,**d**), mean (**e**,**f**) pressure, and heart rate (**g**,**h**) variations in mice receiving standard diet (SD), high fat diet (HFD), and HFD supplemented with apple for 14 weeks. The data were expressed as the mean ± SEM. * *p* < 0.05, ** *p* < 0.01, *** *p* < 0.001.

HFD (Figure 4a,b) increased systolic, diastolic, and mean blood pressure (Figure 4a,c,e). The supplementation with GSCC decreased systolic, diastolic, and mean blood pressure, which became significant at month 2 (Figure 4b,d,f). The supplementation with GSXO reduced systolic blood.

Pressure during the first month, then systolic blood pressure increased being not significantly different from that of HFD mice. Additionally, supplementation with GSXO decreased diastolic blood pressure during the first and second months, and then it increased this parameter until the end of treatment. As consequence, mean blood pressure was decreased during the second month (Figure 4b,d,f).

HFD did not affect heart rate compared to SD group (Figure 4g). Mice fed with the supplementation of GSCC but not GSXO decreased heart rate during the first two months (Figure 4h).

### 3.6. Effects of Apple Supplementation on Atherosclerotic Lesion

**Figure 5:**
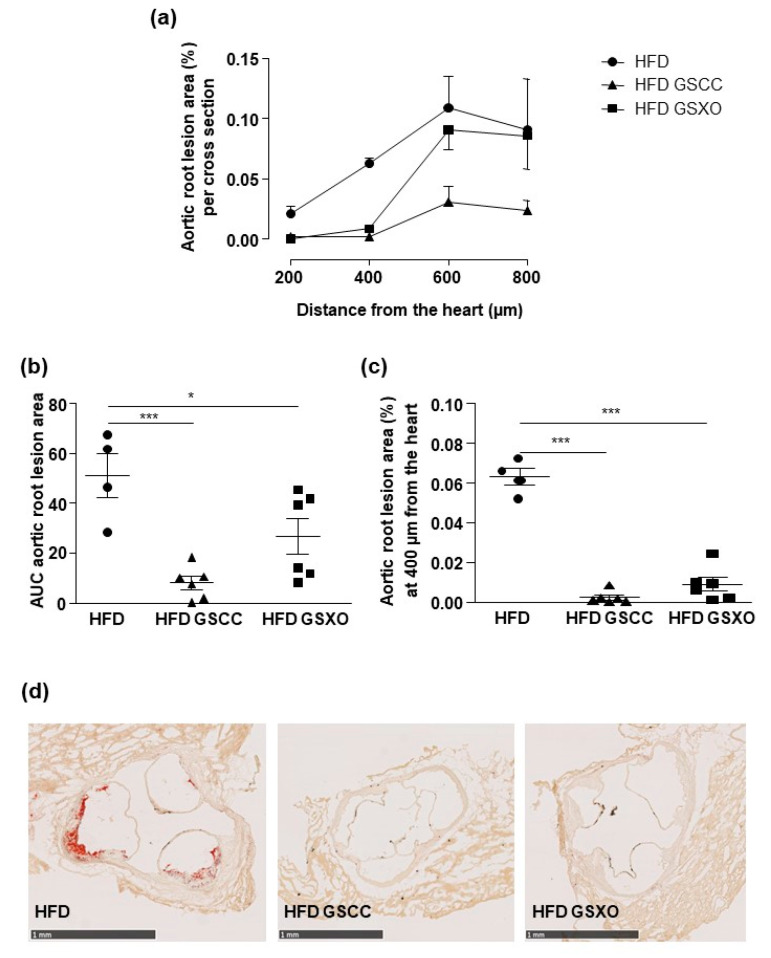
Effect of diet and apple supplementation on atherosclerotic lesions of ApoE-/- mice**.** Extent of atherosclerosis in the aortic root per cross section (**a**), quantification of AUC of aortic root area (**b**), atheroma area at 400 µm from the heart (**c**), and representative images (scale bar 1mm) of stained aorta (**d**) from mice receiving standard diet (SD), high fat diet (HFD), and HFD supplemented with apple for 14 weeks. The data were expressed as the mean ± SEM. * *p* < 0.05, *** *p* < 0.001.

The percent of aortic root lesion area in HFD group increased with the distance from the heart from 200 to 800 µm. The maximum increase was obtained at 600 µm. Mice supplemented with GSCC displayed atherosclerotic lesion only at 600 µm distance from the heart with a level identical to that of nontreated animals. GSXO induced both a rightward shift of the distance of atherosclerotic lesion and reduced the area of aortic lesion (Figure 5a). This was confirmed by the comparison of AUC (Figure 5b). In fact, GSCC and GSXO supplementations decreased significantly the area of lesion compared to HFD group. Both apple supplementations reduced the lesion area at 400 µm (Figure 5c,d).

## 4. Discussion

The present study demonstrates that supplementation with apple flesh from GS variety preserved under two different conditions (classic cold conditions and extreme ultra-low oxygen conditions) prevents most of the metabolic and cardiovascular disturbances in a mouse model of atherosclerosis. Indeed, both GSCC and GSXO decrease circulating TG, and the expression of genes that promote hepatic de-novo lipogenesis, without change in circulating glucose and cholesterol concentrations as well as HOMA-IR. In addition, GSXO supplementation ameliorates body weight gain, insulin level, and HDL/LDL ratio. Most importantly, apple supplementation improves hemodynamic parameters. Supplementation with GSCC decreases systolic, diastolic, and mean blood pressure, after 2 months, in association with cardiac output amelioration. Supplementation with GSXO improves blood pressure during first month of treatment. Finally, supplementation with GSCC and GSXO decreases the area of atherosclerotic lesions in the aortic root. Altogether, these results underscore beneficial hemodynamic and metabolic effects of apple flesh intake whatever the mode of storage in a model of cardiovascular and lifestyle-related cardiometabolic diseases.

Previous report of the Scalbert group [18] using apple polyphenol and fibers extracts alone or in combination using ApoE-/- mice under normal diet, clearly show that under their experimental conditions apple fibers and polyphenols may play a role in preventing atherosclerosis diseases mainly by decreasing uric acid plasma levels. However, the strategy used in the present study is quite different and is based on our recent multiparametric screening on cellular models involved in cardiometabolic diseases showing that apple flesh from GS variety exhibits vascular tropism [10]. Notably, we evaluated the effect of either GS supplementation under the two storage conditions (i.e., CC and XO) in a model of cardiovascular and lifestyle-related cardiometabolic diseases, the ApoE-/- mice fed with high fat diet. In addition to the attenuation of atherosclerosis, we underscore beneficial hemodynamic and metabolic effects of apple flesh intake.

In accordance with other reports [19,20], HFD induces body weight gain in association with the increased of plasma TG level and decreased HDL/LDL ratio. The consequences of the HFD are also evident at hepatic and cardiovascular level. In agreement with previous studies [20,21,22,23], we evidence liver steatosis and modest fibrosis, increased systolic and diastolic blood pressure concomitantly with enlarged atherosclerotic lesion area compared to control mice.

**Figure A1:**
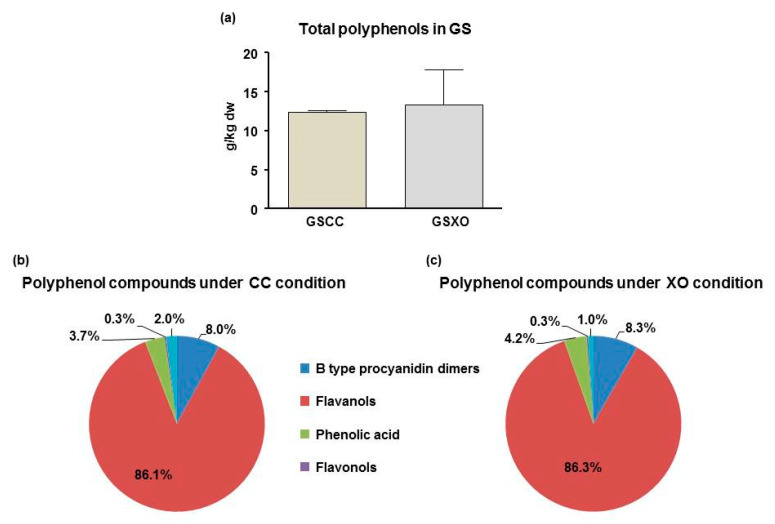
Polyphenol content in apple samples. Histograms represented content of (**a**) total polyphenols, expressed as g/100 kg of dried weight (dw) fruit and the pie charts (**b**,**c**) represented the percentage of compounds found in the samples: flavanols, B type procyanidins dimers, phenolic acid, dihydrochalcones, flavonols in different GS variety under CC and XO conditions.

The storage conditions used in the present study might affect differentially the content, bioavailability, and quality in micronutrient compounds and phytochemicals of GS supplementation [24]. Although storage conditions represent an important determinant of apple fruit quality as many biochemical changes occur, several studies indicate that, as a consequence of storage, the phenolic content and individual polyphenols remained relatively stable or changed slightly [25,26,27]. Accordingly, total polyphenols, as well as the five major groups of polyphenol compounds identified in the samples used in the present study (flavanols, B type procyanidin dimers, phenolic acid, dihydrochalcones, and flavonols) are comparable in CC and XO conditions (Figure A1). Thus, differential effects detected in the present study cannot be explained by changes in polyphenol composition. Nevertheless, they could be attributable to the modifications and/or combinations of other nutritional apple components including vitamins, minerals proteins, carbohydrates, and fibers or other polyphenols not measured in this study.

In the present study, GSXO but not GSCC supplementation significantly decreases the body weight gain induced by HFD. As discussed above this difference is not due to the polyphenols analyzed but might be attributable to other components of apples associated with the storage conditions. Further studies are needed to sort out these differences. Data obtained with GSXO are in line with those reported in middle-aged hypercholesterolemic overweight women, where apple intake has been associated with weight loss after 12 weeks of follow-up [4]. In contrast, another study [18] shows that apple polyphenols and apple fibers administered separately or in association to ApoE-/- mice for 4 months are not able to modify the mouse body weight, as observed with the GSCC supplementation.

The present study shows that GSCC and GSXO supplementations display lipid lowering properties inasmuch they reduce circulating TG and the expression of genes that promote hepatic de-novo lipogenesis, without change in glucose and cholesterol concentrations as well as HOMA-IR. Some animal studies suggest that in rats fed with apple there is a significant improvement of the serum lipid profile [28]. Apart from polyphenols content, pectin has been reported to decrease the total cholesterol, LDL-cholesterol, and TG in obese male Wistar rats [29]. Interestingly, our results are consistent with those from human study in which apple intake in obesity contest, reduces serum triacylglycerols and total cholesterol without modification in glucose:insulin ratio [4].

With regard to hepatic regulation of lipid metabolism associated with hepatic de-novo lipogenesis, we found that GSCC and GSXO decrease the mRNA expression level of *Scd1*, without affecting mRNA expression of other lipogenic enzyme genes (*Dgat1, Srebp1c, Mttp,* and *Lpl*). In addition, GSXO supplementation further decreases the mRNA level of the *Fas* gene in HFD mice. For *FAS*, the apple polyphenol phloridzin has been reported to decrease the activity of hepatic *FAS* in a mouse model of obesity [30]. *Scd1* represents an important metabolic control point. Mice with a disruption in the *Scd1* gene have increased energy expenditure, reduced body adiposity, and increased insulin sensitivity, and are resistant to diet-induced obesity [31]. Thus, we show that reduced hepatic *Scd1* and *Fas* might participate in the beneficial regulation of lipid metabolism by GS supplementation.

We observe that apple supplementation in the context of HFD increases hepatic very low density lipoprotein (VLDL) secretion and, in GSXO group it is associated with hepatic steatosis and modest fibrosis. This is likely due to an increased hepatic VLDL-TG secretion rate similar to that observed in subjects with high intrahepatic TG content [32]. Moreover, accumulating evidence suggests that liver steatosis is a reversible and relatively benign metabolic condition and play a compensatory mechanism at the onset of the disease [33,34]. Nevertheless, we report here that GS supplementation reduces circulating TG and decreases lipogenesis gene expression without irreversible consequence on fat liver accumulation under the experimental condition used.

The most important findings of the present study are that GSCC and GSXO induce a significant reduction in systolic, diastolic, and mean arterial blood pressure with moderate or no effect of heart rate, respectively. These results demonstrate that apple flesh GS exerts an antihypertensive effect in mouse model of atherosclerosis. Our results support data obtained from a large-scale prospective cohort of middle-aged and older women in which a greater intake of apples is associated with lower risks of developing hypertension [35]. This is of importance inasmuch the antihypertensive effects of apple flesh GS are essential to correct vascular remodeling comprising vascular hypertrophy and the associated fibrosis. All of these effects lead to increased vascular stiffness and subsequent vascular dysfunctions. Moreover, the antihypertensive effect in association with lipid lowering properties of apple flesh GS reported in the present study also participates in the reduced atherosclerotic lesion resulting from remodeling of vascular cells including endothelial and smooth muscle cells (see below). Indeed, our previous in vitro study [10] we demonstrated that apple samples exhibited a vascular tropism. In particular, we found that GSCC decreases both oxidative stress and apoptosis in endothelial cells concomitantly with a decrease of smooth muscle cell proliferation; while GSXO increases proliferation of endothelial cells in association with the reduction of apoptosis in same cells and oxidative stress in smooth muscle cells. Thus, GSXO and GSCC are mainly effective on endothelial and smooth muscle cells [10]. These effects probably participate in the antihypertensive effect of GSCC and GSXO reported in the present study.

With regard to atherosclerosis, both GSCC and GSXO increase the distance of atherosclerotic lesion from the heart and reduce the area of aortic lesion reduction in HFD mice. Thus, they exert potent anti-atherosclerotic properties probably due to their effects on different vascular cell types including their ability to reduce oxidative stress of both endothelial and smooth muscle and to increase endothelial proliferation and to reduce smooth muscle cells proliferation in vitro [10]. Our results are consistent with a previous study on ApoE-/- mice under normal diet in which apple constituents supplied at nutritional doses limit the development of atherosclerotic lesions in the aortic sinus mainly by decreasing uric acid plasma levels [18]. Thus, we highlight the novelty of the present study that antihypertensive effect in association with lipid lowering properties of apple flesh GS participates in the reduced atherosclerotic lesion resulting from remodeling of vascular cells including endothelial and smooth muscle cells.

With regard to GS, constituents that support the antihypertensive and anti-atherosclerotic properties include polyphenols and fibers. With regard to polyphenol content, we reported that GS apple flesh has the highest content of total polyphenols compared to other apple varieties such as Gala, Golden Delicious, or Pink Lady. Among polyphenol compounds, GS is rich in flavanols and procyanidin B but it also contains dihydrochalcones and likely flavonols [10]. Among flavonols, quercetin contained in apple has been shown to induce a progressive dose-dependent and sustained reduction in blood pressure when given chronically in several rat models of hypertension. Quercetin is also effective in reducing blood pressure in rat models of metabolic syndrome, including the obese Zucker rats as well as rats treated with a high sucrose, high fat diet [36]. Quercetin and mainly its metabolites also prevent morphological and functional changes in vessels with their ability to reduce reactive oxygen species via scavenging of superoxide anions and inhibition of NADPH oxidase, to decrease vascular smooth muscle cell proliferation and to induce vasodilatation via endothelium-independent and dependent mechanisms and finally to inhibit the angiotensin converting enzyme activity [36,37,38,39].

GS is rich in procyanidin B and procyanidins have been shown to cause a regression of atherosclerosis in a rabbit model and its anti-atherosclerotic efficacy is independent of a cholesterol-lowering effect [40]. Our results are in accordance with those of Xu et al. [41] showing a beneficial effect of apple polyphenols in preventing plaque formation in ApoE-/- Western diet-fed mice by regulating the expression of genes that are involved in lipid metabolism and antioxidant defense. This effect is associated with the ability of apple polyphenols to prevent ox-LDL-induced MAPK/NF-kB activation and to reduce the subsequent endothelial inflammation, which are early critical steps in the formation of arteriosclerotic lesions [41]. Finally, another polyphenol contained in apple such as chlorogenic acid potently reduces atherosclerosis development in ApoE-/- mice due to its hypolipidemic, anti-inflammatory, and antioxidant properties [42].

Altogether, GSCC and GSXO apple flesh exert both antihypertensive activities and reduce atherosclerosis due to their lipid lowering properties; hepatic regulation of lipid metabolism associated with hepatic de-novo lipogenesis and their ability to improve hemodynamic parameters in a mouse model of cardiometabolic diseases. Our present study provides strong evidence that apple GS may be of therapeutic benefit in the future and may represent a new class of nutritional products against obesity-associated diseases.

## Figures and Tables

**Table 1:** Composition of diets used in the present study.

Diet	Standard Diet	High Fat Diet	High Fat Diet+Granny Smith (GS)
**Composition, g/kg**			
GS CC and XO	absent-	absent	3.33
Sucrose	207	340	340
Dairy butter	50	200	200
Casein	200	180.5	180.5
Pregelatinized cornstarch	400	145	145
Premixture of minerals	70	70	70
Crude cellulose	absent	50	46.77
Premixture of vitamins	10	10	10
DL-methionine	3	3	3
Cholesterol	absent	1.43	1.43
**Energy, %**			
Protein	19.3	17.7	17.7
Fat	8.4	41.7	41.7
Carbohydrate	72.4	40.6	40.6

**Table 2:** Effect of diet and apple supplementation on glucose and lipid parameters.

	SD	HFD	HFD GSCC	HFD GSXO
**Glucose (g/L)**	1.62 ± 0.29	1.81 ± 0.34	1.21 ± 0.15	0.92 ± 0.15
**Insulin (g/L)**	0.79 ± 0.09	0.58 ± 0.03	0.89 ± 0.19	1.19 ± 0.17 ##
**HOMA-IR**	0.07 ± 0.02	0.05 ± 0.01	0.05 ± 0.01	0.04 ± 0.01
**TG (g/L)**	0.89 ± 0.22	1.34 ± 0.17 *	0.86 ± 0.10 #	0.56 ± 0.14 ##
**Cholesterol (g/L)**	7.22 ± 1.54	7.70 ± 2.25	8.43 ± 1.22	6.02 ± 1.00

Circulating levels of glucose, insulin, triglycerides (TG), total cholesterol, and homeostasis model for insulin resistance (HOMA-IR) evaluated in fasting plasma mice receiving standard diet (SD), high fat diet (HFD), or HFD containing GSCC and GSXO for 14 weeks. The data were expressed as the mean ± SEM. * *p* < 0.05 vs. SD, # *p* < 0.05, ## *p* < 0.01 vs. HFD.

**Table 3:** Cardiac function following 14 weeks of diets.

	SD	HFD	HFD GSCC	HFD GSXO
**LVESD (mm)**	2.3 ± 0.1	2.3 ± 0.1	2.6 ± 0.1	2.6 ± 0.1
**LVEDD (mm)**	3.4 ± 0.1	3.6 ± 0.1	3.9 ± 0.1	3.7 ± 0.1
**LVESV (mL)**	19.7 ± 2.2	19.4 ± 2.2	25.6 ± 4.1	26.4 ± 2.7
**LVEDV (mL)**	60.6 ± 4.8	55.3 ± 2.4	68 ± 5	60.5 ± 4.2
**Stroke volume (mL)**	40.9 ± 3.6	35.9 ± 2.4	42.3 ± 3.4	34.1 ± 2
**Ejection fraction (%)**	67.2 ± 3.0	65.1 ± 3.6	62.9 ± 3.7	57 ± 2.3
**Shortening fraction (%)**	37.1 ± 2.2	35.5 ± 2.7	34 ± 2.6	29.5 ± 1.6
**Cardiac output (mL/min)**	21.3 ± 4.1	17.4 ± 2.5	27.7 ± 3.6 ##	23 ± 4

The table shows the left ventricular end-systolic diameter (LVESD), left ventricular end-diastolic dimension (LVEDD), left ventricular end-systolic volume (LVESV), left ventricular end-diastolic volume (LVEDV), stroke volume, ejection fraction, shortening fraction, and cardiac output of mice fed with standard diet (SD), high fat diet (HFD), and HDF supplemented with GSCC and GSXO for 14 weeks. The data were given as the mean ± SEM. ## *p* < 0.01 vs. HFD.

## Abbreviations

ANOVA	Analysis of variance
ApoE-/-	Apolipoprotein E-deficient mice
CC	Classic cold condition
DGAT2	Diacylglycerol O-acyltransferase 2
FAS	Fatty acid synthase
GS	Granny Smith
GSCC	Granny Smith variety after storage in classic cold condition
GSXO	Granny Smith variety after extreme ultra-low oxygen condition
HDL	High-density lipoprotein
HFD	High fat diet
HOMA-IR	Homeostasis model for insulin resistance
LDL	Low density lipoprotein
LPL	Lipoprotein lipase
LVEDD	Left ventricular end-diastolic dimension
LVEDV	Left ventricular end-diastolic volume
LVESD	Left ventricular end-systolic diameter
LVESV	Left ventricular end-systolic volume
MTTP	Microsomal triglyceride transfer protein
SCD1	Stearoyl-coenzyme A desaturase-1
SD	Standard diet
SEM	Standard error of the mean
SREBP1C	Sterol regulatory element-binding transcription factor 1
TG	Triglycerides
XO	Extreme ultra-low oxygen condition

## Appendix A

Polyphenol composition of GS

The total polyphenol content in flesh apples of GS under CC and XO conditions is similar (Figure A1a). Five major groups of polyphenol compounds identified in GS sample under both storage conditions including flavanols, B type procyanidins dimers, phenolic acid, dihydrochalcones, and flavonols were comparable as expressed in percentages (Figure A1b,c).
